# Ruminant Salivary Microbes: Passenger or Player in the Rumen?

**DOI:** 10.3390/microorganisms11102390

**Published:** 2023-09-25

**Authors:** Joan E. Edwards, Eun Joong Kim, David R. Davies, Radwa Hanafy, Alison H. Kingston-Smith

**Affiliations:** 1 Institute of Biological, Environmental and Rural Sciences, Aberystwyth University, Gogerddan Campus, Aberystwyth SY23 3EE, UK; ejkim2011@knu.ac.kr (E.J.K.); dave.silage@gmail.com (D.R.D.); 2 Department of Chemical and Biomolecular Engineering, University of Delaware, Newark, DE 19716, USA; radwa@udel.edu

**Keywords:** bolus, bacteria, colonisation, ruminal, oral, saliva

## Abstract

Sampling of ruminant saliva has gained interest as a non-invasive proxy for exploring the structure of the rumen microbiome. However, the subsequent data analysis assumes that bacteria originating from the oral cavity are merely passengers in the rumen and play no active role. In this study, it was hypothesised that metabolically active oral bacteria present in the salivary microbiome play a role in the ruminal degradation of plant material. In vitro cultivation-based enumeration confirmed that the ruminant oral cavity harbours a significant number of anaerobic and cellulolytic bacteria that are metabolically active under ruminal conditions. Bacterial 16S rRNA gene profiling of in vitro enrichments also confirmed that oral-derived bacteria were capable of colonising plant material. Preliminary analysis of the colonising bacteria indicated that bacteria belonging to the genus *Streptococcus* were of particular interest. In conclusion, the findings of the current study clearly indicate that bolus-associated bacteria have the potential to play a metabolically active role in terms of ruminal colonisation and the degradation of plant material. This evidence confirms the merit of the hypothesis that the metabolically active oral bacteria present in the salivary microbiome may play a role in the ruminal degradation of plant material.

## 1. Introduction

Saliva plays an important role in regulating the wateriness and outflow of rumen contents, as well as providing salts for buffering rumen pH [1]. As well as minerals, the saliva contains additional components including urea, ascorbic acid, antibodies, proteins, and other bioactive factors [1,2,3]. Ruminant saliva and its microbe free component have been recently shown to modulate rumen fermentation in vitro [4]. Salivary antibodies (IgA) may play a role in this due to the host-based selection of the rumen microbiome [3]. As a consequence, they are also of interest as a potential tool to selectively manipulate the rumen ecosystem [3,5,6].

In recent years, there has been a growing interest in studying the diversity and community structure of the microbiome present in ruminant saliva. This is primarily because the salivary microbiome has been shown to be valuable as a non-invasive proxy of the rumen microbiome [7,8]. This is due to the process of rumination, where previously ingested feed present in the rumen is regurgitated to the mouth, facilitating further particle size reduction [9]. Rumination events occur regularly over the course of the day, following bouts of feed intake as well overnight and during periods of fasting [10,11]. As previously outlined by Kittelmann et al. [8], there are numerous benefits associated with using saliva as a non-invasive proxy of the rumen microbiome. However, there are also several limitations, which means that salivary microbiome data need to be interpreted cautiously when used as a proxy for the rumen microbiome.

Buccal swab samples contain rumen microbes as well as microbes from the oral cavity. As the environmental conditions of the rumen and oral cavity are very different, it is intuitive that the growth of most oral-associated microbes in the rumen is not optimal. This results in the existence of microbial communities that are specific to these respective environments. As such, when processing salivary microbiome data as a proxy for the rumen microbiome, ideally, oral-associated microbes should be removed from the dataset so that only ‘true’ rumen taxa are left. To date, this has been accomplished bioinformatically via the depletion of the oral taxa identified using machine learning methods or filtering approaches based on arbitrary cut-offs or prior knowledge [8,12]. The timing of buccal sampling relative to feeding can also have a significant impact on the value of the salivary microbiome as a proxy for the rumen microbiome [12]. This is presumably due to changing the relative amounts of rumen-to-oral taxa in the dataset as a result of subsequent feeding or simply increased time since the last rumination event. With buccal sampling, DNA yield can also be low and less reliable in yielding PCR amplification products (particularly for anaerobic fungi) as well as potentially missing taxa with an average relative abundance < 1% [8].

The bacterial taxa in buccal swabs may be biased relative to their ability to represent rumen contents, as it has been reported that they have a greater similarity to the microbiota present in rumen solids than in rumen liquids [12]. This observation led Young et al. [12] to suggest that during the mastication of regurgitated boli, bacteria from rumen solids may adhere to oral mucosal surfaces. Interestingly, it has been previously reported that, based on quantitative PCR, buccal swab and rumen samples have similar bacterial abundances [7]. As such, it is equally plausible to suggest that oral bacteria, if they are metabolically active under ruminal conditions, may be capable of colonising and fermenting ingested plant material in the rumen. If this is the case, then a simple classification of bacteria in the salivary microbiome of ruminants as being ‘oral’ or ‘rumen’ is fundamentally flawed.

Consistent with this concept, a preliminary study by Davies et al. [13] demonstrated that bolus material generated from forage (captured at the oesophageal/rumen aperture (cardia) of cattle) had an anaerobic bacterial population that was capable of fermenting plant material. Despite the viable counts of anaerobic bacteria on the bolus material being 1000-fold less than the average present in the rumen, the bolus-associated microbes still had 32% of the fermentation activity measured in the rumen-fluid-inoculated control [13]. Based on this information, it was hypothesised that metabolically active oral bacteria present in the salivary microbiome may also play a role in the ruminal degradation of plant material and are not simply a passenger.

In order to generate data to substantiate this hypothesis, a series of experiments were performed based on the use of bolus material generated from forage as a means to sample the oral microbiome. Firstly, the amount of total viable anaerobic bacteria and cellulolytic bacteria present on bolus material collected during a prolonged bout of feeding (after animals were fasted overnight) was quantified. Secondly, the ability of the bacteria associated with the bolus material to colonise the plant material was then subsequently assessed using an in vitro enrichment approach. Enrichments were performed both in the presence and absence of rumen microbes, and the resulting bacteria colonising the plant material were analysed using a cultivation independent approach.

## 2. Materials and Methods

### 2.1. Animal Management and Bolus Collection

Experiments were conducted under the authority of licenses under the U.K. Animal Scientific Procedures Act, 1986. Bolus donor cows were non-pregnant and non-lactating Holstein-Friesian dairy cows that had previously been prepared with rumen cannulae (Bar-Diamond, Parma, ID, USA).

Animals were fasted overnight prior to collection of freshly ingested bolus material as previously described [14]. Cows were placed in stalls and their rumen contents partially removed to facilitate collection of the freshly ingested bolus material. Fresh forage harvested from ungrazed field plots (cut on the morning of the experiment) was then offered to the cows, and the generated feed boli were captured at the cardia. After all of the required bolus material was generated, the removed rumen contents were then replaced. The maximum duration of the whole procedure was 30 min.

### 2.2. Quantification of Total Viable Anaerobic Bacteria and Cellulolytic Bacteria Associated with the Formation of Bolus Material

The total viable anaerobic bacteria and cellulolytic bacteria associated with sequentially generated bolus material were quantified and compared with those present on the fresh forage (timothy grass, *Phleum pratense*) that was offered to each cow during bolus generation. Two cows were used to generate the bolus material using the method described above (see Section 2.1), with the bolus collection (1st, 25th, 50th, and 75th bolus) repeated on three consecutive days.

Individual boli from each cow were processed for enumeration of total viable anaerobic bacteria and cellulolytic bacteria immediately after collection using the most probable number (MPN) method of Dehority et al. [15]. Samples (5 g) of bolus or forage (as a negative control) were placed in a bag, which was gassed extensively with carbon dioxide prior to anaerobic diluting solution [16] (45 mL) being added. The bag contents were then stomached for 3 min prior to serial dilution and inoculation into MPN broth for simultaneous total anaerobic bacteria and cellulolytic bacteria counts [15]. After incubation for 21 days at 39 °C, cellulolytic bacteria numbers were estimated via visual degradation of cellulose filter paper (assessed by the breaking up of the paper upon agitation of the tube), and total anaerobic bacteria numbers were estimated using the decrease in pH. The remainder of the bolus sample and harvested fresh forage was processed for dry matter determination via freeze drying.

### 2.3. In Vitro Enrichment of Bolus-Associated Bacteria That Colonised Forage

An in vitro experiment was conducted to enable enrichment of bolus-associated bacteria that were able to colonise fresh forage (perennial ryegrass, *Lolium perenne*) under ruminal conditions. In order to allow comparison of the enriched bacteria from the different potential sources (i.e., plant epiphytes, the rumen, and the bolus generation process), bolus samples and unmasticated forage were both incubated in the presence and absence of a rumen microbial inoculum.

Bolus material from four cows was studied within the experiment, and all incubations for each cow were conducted in triplicate. For each cow, rumen contents were sampled prior to rumen emptying and strained through two layers of muslin before being used to prepare a 25% (*v/v*) ruminal inoculum using anaerobic buffer [17]. Bolus material for incubations, collected as previously described in Section 2.1, was then prepared from each cow by pooling three boli after the first five boli generated were discarded. The harvested fresh forage for incubations was then mechanically damaged in order to mimic mastication, as previously described [14].

Samples of bolus material (14 g FW) or unmasticated forage (10 g FW) were then placed in Schott bottles (100 mL). Different amounts of fresh weight were used due to the bolus material having a lower DM content. To the bottles, 80 mL of either anaerobic buffer or a 25% (*v/v*) rumen microbial inoculum (prepared from the same cow as the bolus material was sourced) was then added to the bottles under a stream of carbon dioxide. The bottles were tightly closed after insertion of a butyl rubber gasket into the lid. Bottles were then inverted to mix the contents prior to incubation at 39 °C for 8 h. At the end of the incubation, bottle solids were placed into polyester bags (22 × 9 cm with 40 µm pore size) and squeezed by hand prior to being machine-washed [18] in order to remove non-colonising bacteria. Washed samples were then snap-frozen in liquid N_2_ and stored at −20 °C prior to being freeze-dried and ground for subsequent DNA extraction.

### 2.4. DNA Extraction

DNA from freeze-dried and ground samples (20 mg) was extracted using a FastDNA SPIN Kit for Soil (MP Biomedicals Europe, Illkirch, France). Manufacturer’s guidelines were followed with the exception that the samples were processed for 3 × 30 s at speed 6.0 in a FastPrep instrument (QBiogene, Cambridge, UK), with incubation for 30 s on ice between bead beating. Integrity of the DNA was verified via agarose gel electrophoresis, and the DNA was quantified using a NanoDrop^®^ ND-1000 Spectrophotometer (Thermo Fisher Scientific, Gloucester, UK).

### 2.5. Bacterial 16S rDNA Denaturing Gradient Gel Electrophoresis Analysis

Bacteria colonising the plant material at the end of the in vitro enrichments were profiled using denaturing gradient gel electrophoresis (DGGE) with the primer pair 799F2 and R1401GC targeting the V6–V8 region of the 16S rDNA, as previously described by Edwards et al. [18]. Amplification of the PCR products was verified via agarose gel electrophoresis. PCR products were then separated using 6% (*v*/*v*) polyacrylamide gels with a 30–60% denaturing gradient (100% denaturant consisting of 40% (*v*/*v*) deionised formamide and 7M urea) in a Dcode system (Bio-Rad UK Ltd., Hemel Hempstead, UK). Gels were run initially for 10 min at 200 V prior to electrophoresis for 16 h at 85 V in 0.5× TAE buffer at a constant temperature of 60 °C [19]. Gels were then stained with silver nitrate [20] and scanned using a GS-800 calibrated imaging densitometer (Bio-Rad UK Ltd., Hemel Hempstead, UK). Cluster analysis was performed on the scanned gel images with the software package Fingerprinting (Bio-Rad UK Ltd.) using the Pearson similarity coefficient (with a position tolerance of 0.5% and an optimisation parameter of 1%), and the dendrograms were constructed using an unweighted pair group method with arithmetic mean (UPGMA) approach.

### 2.6. Sequencing and Analysis of DGGE Bands of Interest

DGGE band positions of interest were excised (with a sterile scalpel blade) from the respective DGGE gels, and the DNA was extracted as previously described [21]. The extracted DNA was reamplified via bacterial PCR as described above, except that the GC clamp was not present on the reverse primer (i.e., R1401). After PCR amplification was verified on an agarose gel, the products were cleaned up using a QIAquick PCR purification kit (Qiagen, West Sussex, UK) following the manufacturer’s guidelines. Amplicons were then cloned into *E. coli* using a pGEM-T Easy Vector System (Promega, Southampton, UK). For each excised band, five clones were sequenced using a BigDye Terminator v3.1 Cycle Sequencing Kit (Applied Biosystems, Warrington, UK) on an ABI3130xl DNA sequencer. Cloned sequences generated in this study were submitted to the National Center for Biotechnology Information (NCBI) database.

The sequences were taxonomically classified using SINA version 1.2.12 [22] and the least common ancestor method. The SILVA SSU database release 138 [23] was used for the taxonomic annotation. Phylogenetic analysis was performed for the sequences that belonged to the *Streptococcus* genus. The obtained sequences were aligned to a bacterial reference SSU sequence dataset downloaded from the NCBI nr database using MUSCLE with default parameters and manually refined in Geneious [24]. The generated alignment was then used to construct a maximum likelihood phylogenetic tree in FastTree, using *Lactococcus lactis* as the outgroup. Bootstrap values were calculated based on 100 replicates.

### 2.7. Statistical Analysis

Bacterial counts (log_10_/kg DM) were compared using analysis of variance (ANOVA) assuming a split plot model with animal as the block, sampling time as the whole plot, and days as the subplot. For bolus material data, effects of bolus number on bacterial counts and DM content were partitioned into polynomial contrasts.

## 3. Results

### 3.1. Quantification of Total Viable Anaerobic Bacteria and Cellulolytic Bacteria in Bolus Material

**Figure 1:**
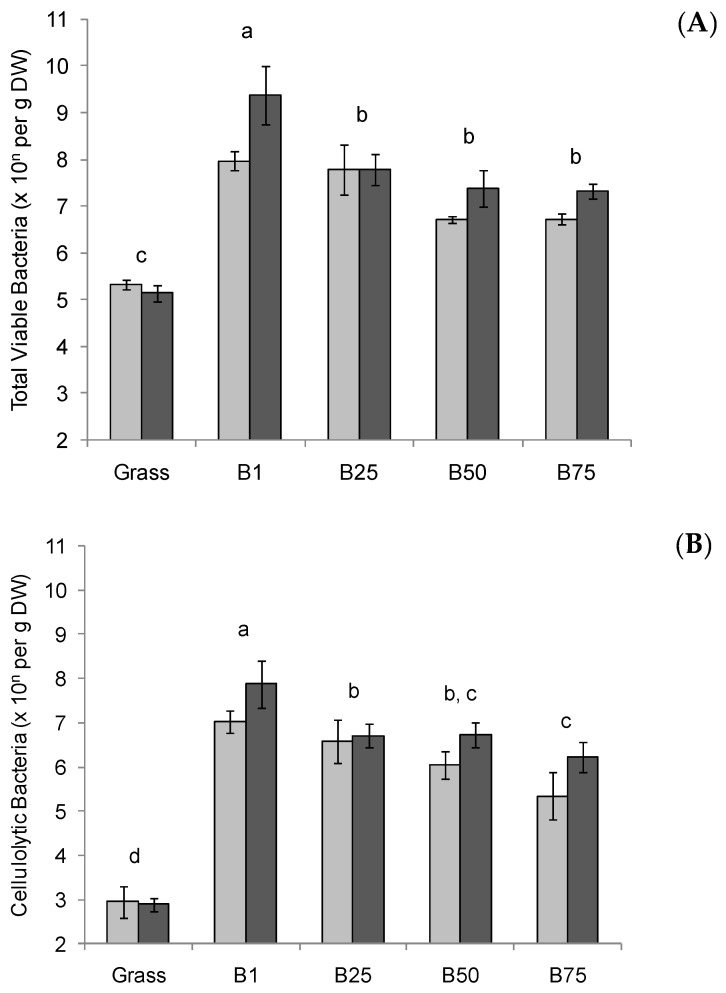
Cultivation-based enumeration of the bacterial microbiota of bolus material. The numbers of total viable anaerobic bacteria (**A**) and cellulolytic bacteria (**B**) were determined for the grass used for bolus generation (Grass) and the bolus material as it was sequentially generated (i.e., B1, 1st bolus generated; B25, 25th bolus generated, etc.). Light and dark grey columns represent the means (n = 3) of boli collected from two different animals, and the error bars the standard error of the mean. As there was no significant effect of animal, lowercase letters above the bars indicate significant differences (*p* > 0.05) over the complete dataset (i.e., n = 6).

For both animals, the concentrations of total viable anaerobic bacteria and cellulolytic bacteria in bolus material are shown in Figure 1, along with those quantified in the grass used for generating the bolus material. The animal used for bolus generation had no effect on the numbers of total viable anaerobic bacteria and cellulolytic bacteria (*p* > 0.05). As such, the statistical analysis of the data was performed using the combined data from both animals on the three consecutive days (i.e., n = 6). Regardless of the stage of bolus collection during the feeding period, the numbers of total viable anaerobic bacteria and cellulolytic bacteria on the bolus material samples were significantly greater (*p* < 0.05) than the numbers found on the grass that was used for the bolus generation (Figure 1).

During the feeding period, significant differences were observed, with the first bolus generated containing more total viable anaerobic bacteria and cellulolytic bacteria than those subsequently collected (*p* < 0.05). A linear (log10 scale) decline in both total and cellulolytic bacterial numbers was noted with increasing bolus number (*p* < 0.001). In contrast to the differences in the amount of bacteria quantified, the dry matter content of the bolus material was consistent throughout the feeding period (7.5%, s.e.m. 0.29) and lower (*p* < 0.05) than that of the grass (14.5%, s.e.m. 0.40) due to the addition of saliva associated with mastication.

### 3.2. In Vitro Enrichment of Bolus-Associated Bacteria Able to Utilise Forage

**Figure 2:**
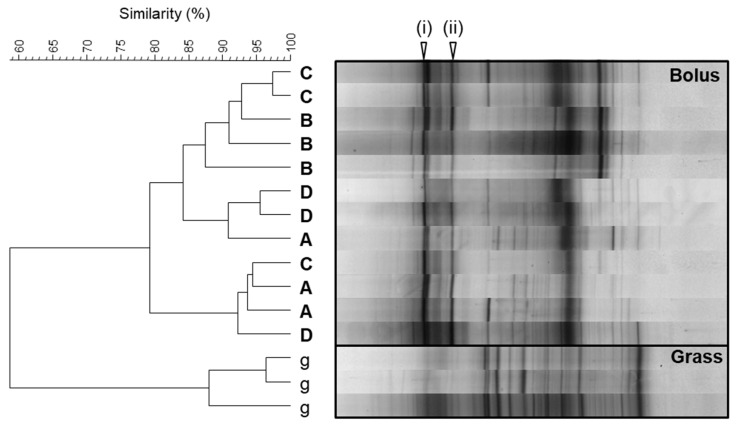
Cluster analysis of DGGE profiles of triplicate samples of bacteria colonising unmasticated forage (g) and bolus material generated from four different animals (A, B, C, and D) following in vitro incubation for 8 h are shown. The arrow heads indicate bands of interest, as described in Section 3.2.

The ability of the bacteria associated with the bolus generation process to colonise the forage was assessed via in vitro enrichment. Collected forage bolus material was directly incubated in buffer under ruminal abiotic conditions (i.e., 39 °C and anaerobic), and the bacterial populations attached to the forage were then subsequently profiled. Unmasticated forage control samples were similarly incubated and profiled in order to account for any enrichment of plant epiphytic bacteria. For all four bolus donor cows, the profiles of bacterial populations colonising the bolus material were found to be distinct from those obtained from the unmasticated forage control (Figure 2). All the bolus samples shared two band positions (i and ii), which were very weak or absent in the unmasticated forage control (Figure 2). Other dominant bands in the bolus samples were either present in the unmasticated forage control or were not consistently observed in all animals.

**Figure 3:**
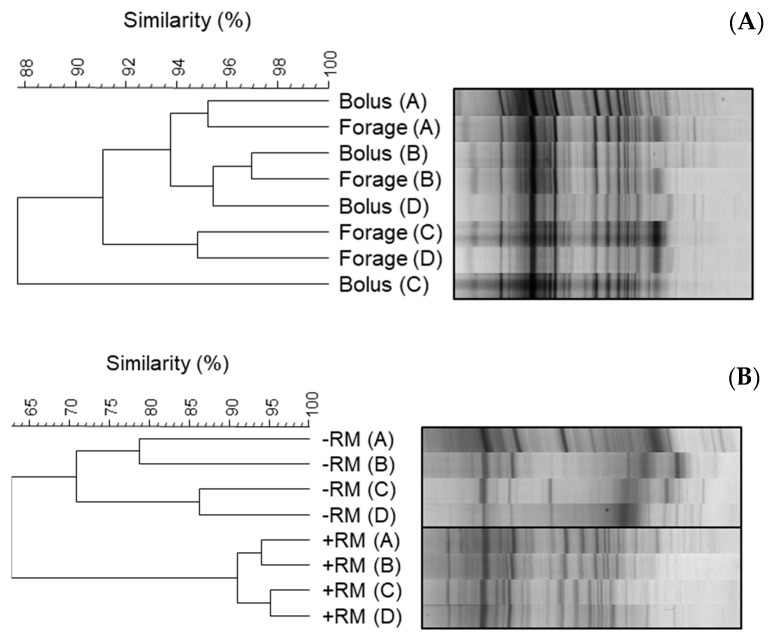
Cluster analysis of DGGE profiles of bacteria colonising bolus material and unmasticated forage following in vitro incubation for 8 h. Comparisons of representative profiles from each animal source (A, B, C, and D) are shown for: (**A**) bolus material (Bolus) and unmasticated forage (Forage) incubated in the presence of rumen microbiota; (**B**) bolus material incubated in the presence (+RM) or absence (−RM) of rumen microbiota.

Parallel in vitro incubations of the bolus material and the unmasticated forage with rumen microbiota were also performed. When incubated with rumen microbiota, the profiles of the incubated bolus and unmasticated forage samples shared a high similarity (>95%) (Figure 3A). In contrast, the profiles generated when bolus samples were incubated with rumen microbiota consistently differed from those generated when bolus samples were incubated with buffer, with the two sample types showing just 65% similarity (Figure 3B).

**Figure 4:**
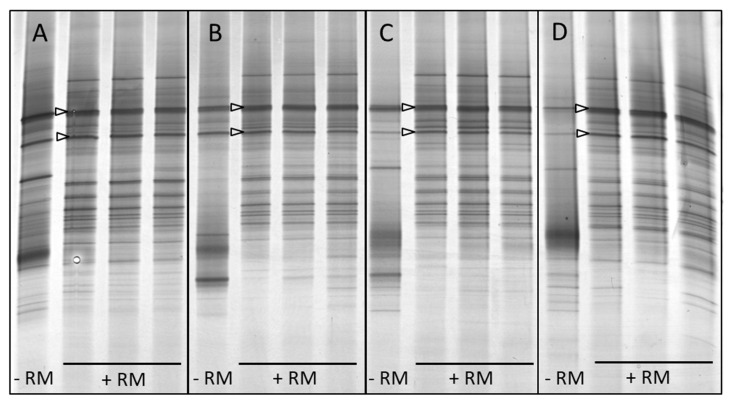
DGGE profiles of bacteria colonising bolus material following in vitro incubation for 8 h in the absence (−RM) or presence (+RM) of rumen fluid. Four separate panels are shown, relating to the four different cows (**A**–**D**) used as bolus and rumen fluid donors. Arrowheads indicate the two band positions (i, upper arrowhead; ii, lower arrowhead) previously indicated in Figure 2.

All bolus samples, whether incubated in the presence or absence of rumen microbiota, had the same two band positions that were previously observed in the buffer-incubated bolus samples (Figure 4). In order to make a preliminary identification of the bolus-associated bacteria representing these, bands were excised from the profiles of the bolus samples incubated without rumen microbiota (−RM profiles, Figure 4), and their DNA was extracted and cloned prior to sequencing. For each band position and animal, five clones were randomly selected for sequencing (i.e., 2 band positions × 4 animals × 5 clones = 40 clones). Sequences annotated as belonging to the genus *Streptococcus* were most abundant in the dataset (67.5%) and were associated with both band positions in each of the four cows (Table 1). The remainder of the sequences represented taxa that were only found once.

**Figure 5:**
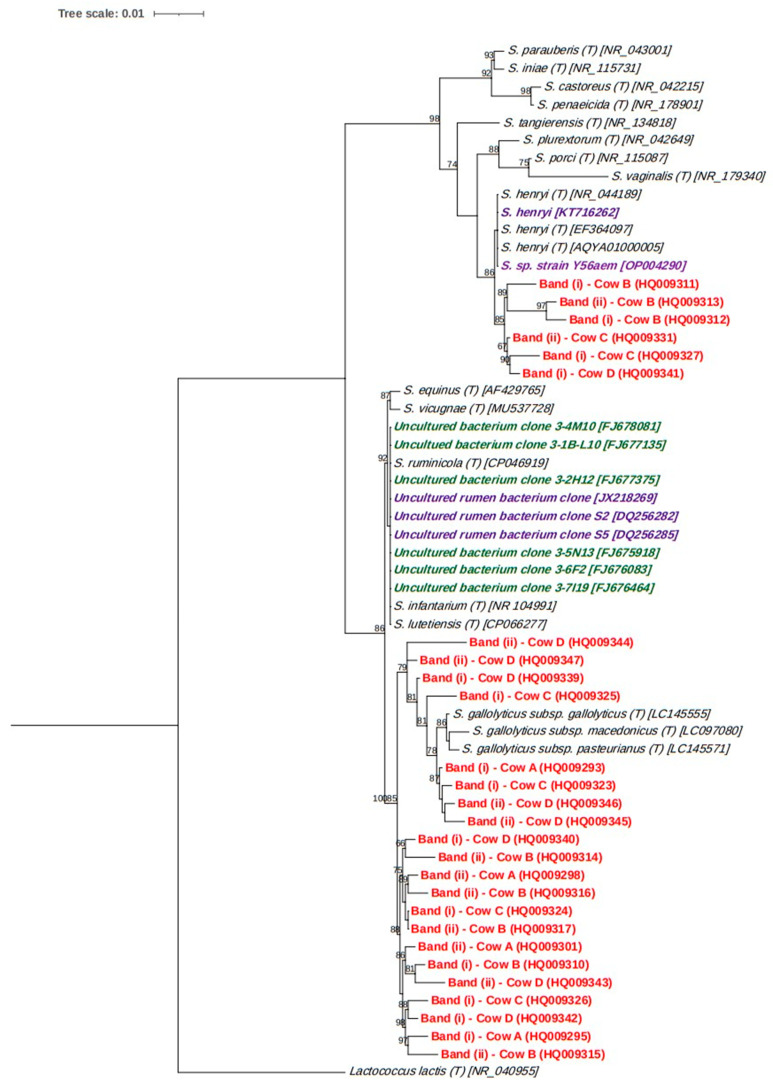
Phylogenetic tree of the cloned *Streptococcus* sequences generated in this study (red). NCBI accession numbers are indicated in brackets for all sequences in the tree. Type strain sequences are indicated by ‘(T)’. Non-type strain sequences are also included in the tree, which were derived from the rumen (purple) or ruminant faeces (green).

In order to provide more insight, a phylogenetic tree was constructed using the *Streptococcus* sequences. The *Streptococcus* sequences fell into two separate sections of the tree (Figure 5). The upper branch of the tree contained six of the study sequences as a monophyletic clade, which was closely related to *Streptococcus henryi*, and, based on a BLAST search, they had 97.6–99.7% identity with the type strain of *S. henryi*. The remaining 21 sequences all clustered within a lower branch of the tree, which further divided into two monophyletic lineages. One of these lineages contained eight of the study sequences along with the type strains for the three subspecies of *Streptococcus gallolyticus* and had >98.5% identity with them. The remaining 13 sequences formed a clade by themselves.

## 4. Discussion

A previous preliminary study indicated that freshly ingested bolus material had an anaerobic bacterial microbiota that had significant fermentative activity [13]. In this study, bolus material was confirmed to contain a large number of metabolically active anaerobic bacteria, even after a prolonged bout of feeding (i.e., bolus generation). Furthermore, it was demonstrated that a large proportion (approx. 20–40%) of these bacteria were cellulolytic. This proportion of cellulolytic bacteria is comparable to that previously reported in the rumen using the same cultivation-based methods as employed in this study [15].

A significant negative linear correlation was noted between the amount of bacteria present in the bolus material and the number of boli generated. This may have been due to the oral bacteria originating from rumination events prior to feeding. However, it is also consistent with the dilution of oral bacteria due to the constant production of saliva during a feeding bout [25]. Regardless of the origin of the bacteria, it is clear from these findings that the bolus material contains bacteria that are metabolically active under in vitro abiotic conditions representative of the ruminal environment.

As the forage down bolus is a compact ball of rolled material [9], cellulolytic oral bacteria may have an ecological advantage. They are already distributed throughout the bolus following ingestion, unlike the rumen bacteria, which would only be able to initially colonise the exterior of the bolus. Rumen bacterial colonisation of ingested plant material is a key stage in its ruminal degradation [26] and occurs within minutes of its ingestion [18,27]. In order to explore if bolus-associated bacteria could colonise plant material, in vitro enrichments were carried out under ruminal abiotic and biotic conditions.

Enrichment of the bolus material and the forage used to generate the boli under ruminal abiotic conditions resulted in profiles of the colonising bacteria that were clearly distinct from each other. Two band positions were present in all of the buffer-incubated bolus samples, and the same band positions were also evident in bolus samples incubated with a rumen microbial inoculum. The preliminary identification of the bacteria representing these two consistent band positions in the buffer-incubated bolus samples was accomplished using the sequencing of the excised DGGE bands. The taxonomic classification of the sequences indicated that *Streptococcus* dominated the dataset and was present in both band positions for all four of the cows used in the study.

Whilst several of the excised bands were exclusively represented by *Streptococcus* sequences, many of the bands contained multiple taxa. The retrieval of multiple sequences from single DGGE band positions is not an uncommon phenomenon [28]. As DGGE typically only detects major bacterial taxa within samples [29], the relevance of the sporadically found bacterial taxa is not clear (particularly considering the limited number of clones sequenced per band). Future use of high-throughput amplicon sequencing (HAS) would circumvent this issue by providing sequence data for all the taxa detected, including more minor taxa (depending on the sequencing depth used). However, HAS and DGGE still have fundamental drawbacks due to both being PCR-based.

Irrespective of the profiling method employed in this study, it is clear that the dominance of *Streptococcus* in the sequence dataset generated from the bolus material is consistent with the findings of other studies that have shown that *Streptococcus* is typical of buccal swab samples. Kittelmann et al. [8] identified that *Streptococcus* operational taxonomic units (OTUs) could account for up to 21% of the bacteria detected in buccal swab samples, in contrast to only up to 0.5% of the rumen samples collected via stomach tubing. Furthermore, Kittlemann et al. [8] showed using phylogenetic analysis that the *Streptococcus* OTUs detected in the buccal swab samples were unlikely to be ruminal in origin. Consistent with this, Young et al. [12] indicated that several different *Streptococcus* OTUs were important in discriminating buccal swab samples from ruminal liquid and solid samples. De Freitas et al. [30] also found that *Streptococcus* was enriched in saliva samples relative to rumen and faecal samples. Conversely, Tapio et al. [7] and Miura et al. [31] did not report *Streptococcus* being present in any of the saliva or rumen samples analysed in their respective studies. The reason for these contrasting findings is not clear.

In this study, preliminary analysis indicated that *Streptococcus* consistently colonised the bolus material and that the associated taxa were phylogenetically diverse. Some of the sequences clustered in a clade close to the facultative anaerobe *S. henryi,* which was first described in the equine caecum [32], although it is clear that this species was also detected in the rumen (Figure 5). The remainder of the sequences were within a separate lineage containing *S. gallolyticus,* which is part of the *Streptococcus bovis/equinus* complex [33,34]. *S. gallolyticus* is a well-known species that is commonly found in the rumen and has the ability to metabolise a broad range of plant carbohydrates, and it shares many properties with two oral *Streptococcus* species [35]. However, based on comparative genomic analysis, it has been suggested that the ability of *S. gallolyticus subsp. pasteurianus* to live in the rumen may be reduced relative to *S. gallolyticus subsp. gallolyticus* [36]. As a consequence, from the preliminary analysis performed in this study, it is not clear if the bolus-associated *Streptococcus* bacteria may be better adapted for survival in the oral cavity than the rumen or that they are equally well suited to living in both environments.

Further research is now needed to identify and isolate bolus-associated bacteria and assess their fitness and phenotype under conditions representative of the oral cavity and rumen. Whilst bacteria belonging to the *Streptococcus* genus should be a key focus, it is clear that the findings of this study also need to be extended to other ruminant animals, for example, by surveying a wider number of animals for oral-associated bacteria that are active under ruminal conditions, including animals fed different diets and belonging to other ruminant species. Ecological studies are then needed in order to understand their role in both environments, and their relative contribution to ruminal feed degradation over time.

In conclusion, the findings of the current study clearly indicate that bolus-associated bacteria have the potential to play a metabolically active role in terms of ruminal colonisation and degradation of plant material. This evidence confirms the merit of the hypothesis that metabolically active oral bacteria present in the salivary microbiome may play a role in the ruminal degradation of plant material.

## Figures and Tables

**Table 1:** Taxonomic classification of the 40 cloned sequences from two different band positions (i and ii) that were excised from representative DGGE profiles of buffer-incubated bolus material originating from four different cows (A–D).

Band Position	Cow	Clone NCBI Accession No.	Taxonomic Classification
**i**	**A**	HQ009293	*Streptococcus*
		HQ009294	Family Lachnospiraceae, genus unclassified
		HQ009295	*Streptococcus*
		HQ009296	*Shuttleworthia*
		HQ009297	*Pantoea*
	**B**	HQ009308	Family Microbacteriaceae, genus unclassified
		HQ009309	*Actinobacillus*
		HQ009310	*Streptococcus*
		HQ009311	*Streptococcus*
		HQ009312	*Streptococcus*
	**C**	HQ009323	*Streptococcus*
		HQ009324	*Streptococcus*
		HQ009325	*Streptococcus*
		HQ009326	*Streptococcus*
		HQ009327	*Streptococcus*
	**D**	HQ009338	*Selenomonas*
		HQ009339	*Streptococcus*
		HQ009340	*Streptococcus*
		HQ009341	*Streptococcus*
		HQ009342	*Streptococcus*
**ii**	**A**	HQ009298	*Streptococcus*
		HQ009299	*Rhodopseudomonas*
		HQ009300	*Clostridium sensu stricto* 1
		HQ009301	*Streptococcus*
		HQ009302	Unclassified
	**B**	HQ009313	*Streptococcus*
		HQ009314	*Streptococcus*
		HQ009315	*Streptococcus*
		HQ009316	*Streptococcus*
		HQ009317	*Streptococcus*
	**C**	HQ009328	*Lactococcus*
		HQ009329	*Citrobacter*
		HQ009330	*Burkholderia-Caballeronia-Paraburkholderia*
		HQ009331	*Streptococcus*
		HQ009332	*Mannheimia*
	**D**	HQ009343	*Streptococcus*
		HQ009344	*Streptococcus*
		HQ009345	*Streptococcus*
		HQ009346	*Streptococcus*
		HQ009347	*Streptococcus*

## Data Availability

The sequence data generated in this study are openly available from the NCBI database, and the associated database accession numbers are provided in Table 1.
